# Towards a genuinely medical model for psychiatric nosology

**DOI:** 10.1186/1741-7015-10-5

**Published:** 2012-01-13

**Authors:** Randolph M Nesse, Dan J Stein

**Affiliations:** 1 Department of Psychiatry, University of Michigan, East Hall Room 3018, Ann Arbor, MI 48109, USA; 2 University of Cape Town, Private Bag X3, Rondebosch 7701, South Africa

## Abstract

Psychiatric nosology is widely criticized, but solutions are proving elusive. Planned revisions of diagnostic criteria will not resolve heterogeneity, comorbidity, fuzzy boundaries between normal and pathological, and lack of specific biomarkers. Concern about these difficulties reflects a narrow model that assumes most mental disorders should be defined by their etiologies. A more genuinely medical model uses understanding of normal function to categorize pathologies. For instance, understanding the function of a cough guides the search for problems causing it, and decisions about when it is expressed abnormally. Understanding the functions of emotions is a foundation missing from decisions about emotional disorders. The broader medical model used by the rest of medicine also recognizes syndromes defined by failures of functional systems or failures of feedback control. Such medical syndromes are similar to many mental diagnoses in their multiple causes, blurry boundaries, and nonspecific biomarkers. Dissatisfaction with psychiatric nosology may best be alleviated, not by new diagnostic criteria and categories, but by more realistic acknowledgment of the untidy landscape of mental and other medical disorders.

## Background

The Diagnostic and Statistical Manual of Mental Disorders (DSM) [1] is the object of unrelenting criticism [2-7]. The first page of a leading psychiatry textbook says, "there is little reason to believe that these categories are valid" [8]. Clinicians say DSM categories ignore important aspects of many patients' problems. Teachers report that reification of diagnoses leads students to neglect important phenomenology [8,9]. Researchers protest requirements to use DSM categories that do not map well to neuroscience hypotheses [10]. Non-professionals are skeptical about disorders defined by committees. And, physicians in other areas of medicine cannot help but wonder why psychiatric diagnosis is so problematic.

The current crisis in psychiatric nosology originated in the solution to a previous crisis. In the early 1970s, psychiatry awoke from a long dream to find itself floating on a couch in the backwaters of medicine. A wake-up call came when a 1973 article in *Science *reported that 12 "pseudopatients," who pretended to hear hallucinations, were hospitalized and received a diagnosis of schizophrenia, even though they acted normal after admission [11]. More positive calls came from psychiatrists reporting remarkably selective responses to new medications [12], from researchers identifying genetic influences [13], and from new proposals for diagnostic criteria [14].

By the late 1970s, psychiatry was eager to establish its scientific credibility, and the unreliability of psychiatric diagnosis was an obvious problem. For instance, the DSM-II definition for Depressive Neurosis was: "an excessive reaction of depression due to an internal conflict or to an identifiable event such as the loss of a love object or cherished possession" [15]. Is depression after loss of a favorite cat "excessive?" One diagnostician would say, "Yes," another, "Obviously not!" Such unreliability made research impossible, and psychiatry's scientific aspirations laughable.

The solution was the DSM-III [16]. Published in 1980, it jettisoned psychoanalytic theory, and replaced clinical impressions with checklists of operationalized indicators. The DSM-III criteria for major depression required the presence of at least five of nine possible symptoms for a duration of at least two weeks. The details changed slightly in the 1994 DSM-IV, (adding criteria requiring clinically significant distress or impairment), but these revisions did not change the core strategy of using checklists of criteria to define diagnostic categories [17].

Operationalized diagnosis transformed psychiatry [18]. It made possible standardized interviews epidemiologists could use to measure the prevalence of specific disorders [19]. Neurobiologists could search for pathology specific to reliably defined conditions. Clinical researchers at multiple sites could collaborate on treatment studies that produced massive datasets, now summarized in treatment guidelines. Regulatory agencies, insurance companies and funding agencies could, and soon did, require DSM diagnoses. Psychiatrists could finally diagnose and treat specific disorders, just like other physicians. The solution to the crisis of the 1970s has, in many respects, succeeded beyond all expectations.

## Problems

Operationalizing diagnosis also revealed major problems. We use the word "revealed" because many problems associated with the revised DSM systems were not caused by it, but were revealed by studies it made possible. Comorbidity was found to be prevalent; most individuals who have one disorder also qualify for additional diagnoses [20,21]. Heterogeneity of patients within diagnostic groups is substantial; for instance, two individuals with no specific symptom in common may both qualify for the diagnosis of major depression. Boundaries separating individuals with and without a disorder appear arbitrary, and they are often not separated by a "zone of rarity" [22]. Finally, with the exception of neurological disorders such as Huntington's Disease, not one of the main DSM mental disorders can be validated by laboratory or imaging biomarkers.

Checklist systems also have been said to encourage superficial evaluations that focus on DSM criteria to the exclusion of other phenomenology and attempts to understand the origins of an individual's problems [9,23]. The introduction to the DSM-IV includes a disclaimer: "In DSM-IV, there is no assumption that each category of mental disorder is a completely discrete entity with absolute boundaries dividing it from other mental disorders or from no mental disorder. There is also no assumption that all people described as having the same mental disorder are alike in all important ways" [24]. However, "cautionary statements within the DSM-IV, if read at all, provide little protection among many communities of users against reification of the disorders listed within [3] p 158." The use of categories based on operationalized criteria encourages thinking about diagnoses as if they are discrete diseases - "essentialized natural kinds" in philosophy's lingo [25,26]. Such reification of diagnoses discourages attempts to understand how multiple factors interact over time to cause a range of unfolding cognitions and emotions in any particular individual [26].

Many medical disorders are defined by a specific etiology, or by distinctive anatomical or molecular abnormalities. Despite exhaustive searches, no comparable objective indicators have been found for any major mental disorder [27]. Some statistically significant neurobiological differences characterize certain diagnostic groups (for example, on brain imaging), but they are neither specific nor sensitive enough to validate any diagnosis. The chair of the DSM-IV Task Force, Allen Frances, notes "the disappointing fact is that not even one biological test is ready for inclusion in the criteria sets for DSM-V" [28]

## Proposed solutions

Several solutions have been considered: revising the criteria, radically reformulating them, using biomarkers to define new categories, and creating new categories based on brain circuits or functions.

DSM criteria are being revised by a 29 member Task Force, which coordinates the work of 6 Study Groups and 13 Work Groups [29]. Their product, the DSM-5, scheduled for publication in 2013, will likely incorporate changes, such as including all psychiatric and other personality disorders on a single Axis, and specifying levels of impairment for personality functioning. It may also combine some categories (for example, substance dependence and substance abuse into substance use disorder) and split others (for example, agoraphobia may become a diagnosis separate from panic disorder). However, the core approach will remain the same: operationalized criteria will define categories that are neither framed by a theoretical understanding of normal function nor validated by objective biomarkers. Few believe the DSM-5 will satisfy the critics of the DSM-IV.

Larger changes have been considered. For instance, committees have considered adding a quantitative dimension to measure the severity of diagnoses objectively, without reference to a "clinical significance" criterion [30,31]. They also considered basing diagnoses on a patient's similarity to prototype diagnoses [32,33]. Most experts have concluded that such major changes would cause confusion and decrease diagnostic consistency, with disadvantages that would outweigh benefits [3,34,35].

Another possible solution is to push harder to find biomarkers that define disorders. This is the main current strategy, as reflected by many articles [10,36,37], and the title of the first chapter of the previously mentioned psychiatry textbook: "Introduction and considerations for a brain-based diagnostic system in psychiatry" [8]. This approach will eventually succeed for some disorders, but three decades of consistently negative results suggest it is time to step back and ask why we cannot find diagnostic biomarkers, and what alternative approaches are available.

Some neuroscientists suggest that better categories may come from studying "brain circuits" [2]. This reflects growing recognition that disorders do not necessarily correspond to pathology in specific brain regions or neurochemicals, and that functions are carried out by pathways that connect diverse loci. This approach justifiably highlights adaptive functions; however, it encourages a potentially misleading analogy of evolved brain systems with human-designed circuits. Circuits designed by engineers have discrete modules with specific functions and defined connections that are all necessary for normal operation. Evolved information processing systems have components with indistinct boundaries, distributed functions, massive redundancy, and innumerable connections that comprise systems very different from anything that an engineer, or a neuroscientist, could even describe exactly [33,38]. These factors may help to explain why neuroimaging, like other putative biomarkers, has relatively low sensitivity/specificity for psychiatric diagnostic categories.

A related initiative proposes Research Domain Criteria (RDoC), with five domains (negative valence systems, positive valence systems, cognitive systems, systems for social processes and arousal/regulatory systems) that intersect with seven units of analysis (genes, molecules, cells, circuits, laboratory findings, behavior and self-reports) [36]. The hope is that RDoC will help identify abnormalities that characterize mental disorders. For instance, RDoC might encourage investigators to group together individuals with heightened amygdala responsiveness, regardless of their diagnoses. This approach has the virtue of trying to understand pathology in a framework based on normal functions, but it remains committed to the hope that most psychiatric diagnoses will eventually be based on biomarkers.

A fundamentally different approach to improved diagnosis has come from evolutionary perspectives. Wakefield's definition of mental disorders as "harmful dysfunctions" has spurred important recognition of the need to consider abnormal functions of evolved systems in the context of social values [39]. Other philosophically and biologically sophisticated articles also propose approaches to diagnosis based on an evolutionary understanding of adaptive functions [40,41]. These ideas provide a valuable connection to the kind of functional thinking that physiology offers to the rest of medicine.

## Learning from the rest of medicine

Reducing concerns about nosology for mental disorders to the level typical for diagnosis in the rest of medicine would be a great advance. Psychiatry has emulated the rest of medicine by seeking causes and categories in biological mechanisms, but because it lacks the kind of functional framework that physiology often provides for the rest of medicine, there is a temptation to conceptualize disorders in an essentialist way that oversimplifies reality. Thus, psychiatry's diagnostic categories have been based on mixtures of tradition, clinical experience and brute empiricism. Despite cautions that such categories must be tentative, they are inevitably reified.

### Emotions are adaptive responses

Physicians in other medical specialties routinely distinguish direct manifestations of bodily malfunction from symptoms that are normal protective responses. Seizures, paralysis and dyskinesias arise from abnormal bodily mechanisms. Cough, pain and fever, by contrast, are normal protective responses shaped by natural selection in conjunction with regulation systems that express them in situations where their benefits are likely to exceed their costs [42,43]. Cough clears foreign material from respiratory passages; patients who cannot cough are likely to die from pneumonia. Pain is useful when tissue is being damaged; patients with congenital absence of pain usually die young. Treatment to relieve cough or pain is prescribed only after investigating what is causing them.

Capacities for anxiety and mood also exist because they offered selective advantages to our ancestors [44-47]. Emotions adjust diverse aspects of physiology, cognition, behavior and motivation in ways that increased ability to cope with situations that influenced fitness during our evolutionary history [48]. Their utility is confirmed by the existence of systems that regulate their expression; such systems could evolve only if the responses were useful in certain circumstances. It is also confirmed by the complications that can arise from blocking normal defenses, such as fast progression of pneumonia after excessive cough suppression.

If defensive responses are normal and useful, how can medications that block them ever be safe? Apparently excessive defense expression can often be explained by the "smoke detector principle." False alarms are common and expected because the costs of expressing a defense is often small compared to the potentially huge costs of failing to respond adequately to a real danger [42]. This principle and redundant protective systems explain why is it often safe to use medications to block normal pain, fever, cough and anxiety.

Defense regulation systems can fail, giving rise to responses that are abnormal in any circumstance. Most defensive responses are aversive, so their inappropriate arousal causes much suffering. High prevalence rates for chronic pain, chronic fatigue, anxiety disorders and depression suggest that the regulation mechanisms underlying cognitive/emotional symptoms are especially vulnerable to failure [42]. Most such failures are not complete but involve responses that are too soon, too strong or too prolonged for the situation. Other failures, such as in bipolar disorder, reflect more fundamental control system abnormalities that can result in oscillations that sometimes leave the system stuck at an extreme.

Recognition that emotions are adaptive responses akin to pain and cough has implications for assessment and treatment. Determining if an emotional response is normal or pathological requires knowledge about whether the situations or internal motivational structures that normally arouse the emotion are present [7,49]. While some conditions, such as recurrent severe major depression, are clearly abnormal, diagnosing an expression of emotion as abnormal without considering the life context is like diagnosing chronic pain without looking for possible causes of tissue damage.

Unfortunately, it is difficult to differentiate normal emotions from those expressed inappropriately. A long tradition of trying to distinguish endogenous from exogenous depression has mostly been abandoned because it is hard to do reliably, and because their symptoms and treatment responses are similar.

Even the most prototypically understandable exogenous depression - bereavement - is the focus of current debate. With the exception of atypical or extreme symptoms, DSM-IV criteria exclude a diagnosis of major depression in the two months after loss of a loved one because depression symptoms are normal in that period. Wakefield and colleagues have suggested expanding exclusion criteria to other extreme situations in order to avoid misdiagnosing normal sadness as pathological depression [50], and they note that narrowing the DSM-IV exclusion criteria actually reduced diagnostic validity [51]. Kendler and others suggest eliminating the grief criterion. They note that the International Classification for Diseases has never had a grief exclusion, that depression arising from bereavement is not clinically distinct from other depression, that having a single exclusion is logically inconsistent, and that expanding exclusions to other situations would cause confusion and decrease reliability [52,53].

Eliminating the grief exclusion would increase consistency and reliability, but at a cost not only to validity, but also to common sense; bereavement is not a mental disorder. Extending the exclusion to other situations would make the diagnosis of emotional disorders more like that in the rest of medicine, where normal responses are distinguished from the problems that arouse them, and where detailed information about function and context is used to consider the possibility that the symptom might arise from an abnormal regulation mechanism.

A recent report finds that 61% of DSM diagnoses include criteria about context [54]; however, few are based explicitly on the kind of functional understanding of normal responses that guides decisions in the rest of medicine. For instance, cough clears foreign matter from respiratory passages, so its presence motivates a search for possible causes; cough itself is considered abnormal only when no elicitor can be found. In psychiatry, emotions sufficient in duration and intensity are categorized as disorders irrespective of the situation. This encourages treatment without investigating possible causes, on the assumption that anxiety and depression are abnormal.

The implication for psychiatric nosology is that emotional states should be classified as disorders only if they are excessive for the situation. Deciding what is excessive requires knowledge about what situations normally arouse the symptom, in conjunction with a search for such situations. This is not a new idea; previous versions of the DSM listed reactive emotional states separately from endogenous conditions that arise from faulty regulation mechanisms, and DSM-IV sometimes requires a judgment based on context (for example, in Adjustment Disorder). What is new is recognizing that emotions serve functions in the same way that pain, cough and fever do, and that they are regulated by mechanisms shaped by natural selection.

There are also differences between emotions and other defenses. Pain, cough and fever are usually aroused by specific identifiable problems. Anxiety, anger and low mood are aroused by situations harder to specify, and less readily characterized as abnormal. For instance, a man who is laid off from a job might feel anxiety about possibly losing his home, anger about the employer's broken promises that may be excessive because it stirs childhood memories, and low mood because he sees no way to find a new job. These responses are not diseases, but they nonetheless pose adaptive challenges that arouse emotional responses, just as pneumonia arouses fever and cough. Some common situations, such as being trapped in an abusive marriage, impair social function as drastically as pneumonia disrupts respiratory function, so it is not surprising that they arouse substantial symptoms. This does not imply that such emotions are usually useful in the individual instance, any more than experiencing pain is usually useful; it means only that they are adaptive capacities shaped by selection.

Like other symptoms, emotions arise from interactions of persons with situations. Clinical assessments understandably focus on trait differences among individuals; most anxious patients are concerned about their life-long tendency to excess anxiety, not about their reaction to a particular spider. However, changing a patient's emotional experience often requires close attention to the current motivational structure of his or her life. This includes the person's goals, strategies, opportunities and obstacles in each area. Unfortunately for research, motivational structures involve idiosyncratic values and psychological characteristics interacting with a lifetime of experiences and the current situation. Clinicians intuitively recognize how some situations give rise to symptoms; anxiety is understandable in a woman whose husband's tennis partners have all recently left their wives for younger women. Finding ways to code such data and bring it into a biological framework is a challenging, ongoing project.

Recognizing aversive emotions as adaptive responses may help challenge oversimplified conceptualizations of psychiatric disorders. Like pain and fever, anxiety and depression are nonspecific symptoms that can be aroused by many different problems, so comorbidity and heterogeneity are to be expected. Like the presence of other defenses, the presence of an intense emotion should set in motion a search for situational causes, as well as for individual differences in traits. All emotions are caused by brain changes, but only in the same superficial sense that brain activity in the medulla explains cough. Individual trait differences in emotional responsiveness can arise from brain differences, but they can also arise from differences in cognitive-affective schemas. The complexity of person x's situation interactions frustrates attempts to generalize about causes; the important factors differ from person to person, and even from episode to episode in the same person.

Major challenges confront the project of framing nosology for emotional disorders in terms of the normal functions of emotions. For instance, the DSM has focused on excesses of a few aversive emotions, but disorders of excess and deficit should exist for every emotion; this includes deficits of negative emotions, such as anxiety, and excesses of positive emotions, such as joy. Grouping all emotional disorders together in a category called "Abnormalities of emotion regulation" would make it clear that negative emotional states can be normal, and that information about context is essential to decide whether the expression of an emotion is normal or abnormal.

Deciding how to use information about context is an admittedly large challenge. A simple approach would be to code the causes for each emotional state as None-Mild-Moderate-Severe on two axes, one indicating the Trait Vulnerability, the other the intensity of Current Situations likely to arouse it. Would such coding be practical? While the difficulties would be substantial, the history of medicine documents the value of trying to distinguish clinically similar conditions with different causes, even when that compromises reliability.

To illustrate, consider two cases. A community college student, whose parents and siblings have not experienced mental disorders, functioned well until manifesting typical symptoms of Major Depression in the past three months. An analysis of his motivational structure reveals that he hates being in community college, but feels he must continue or his girlfriend will leave him. She is still in high school, but will soon leave town to attend an elite university in a distant state. Codes of *Moderate *on the Current Situation axis, and *None *on the Trait Vulnerability axis, provide important information for treatment planning. In contrast, another young man with similar symptoms reports being abused by his stepfather after his father, who suffered from depression and alcoholism, left when he was two years old. He has always felt isolated and inadequate, but he has a stable job and several close friends. A diagnosis that includes *Severe *Trait Vulnerability factors, and *Mild *Current Situation factors, would communicate important information about his disorder.

### Syndromes that reflect system failures

Medical conditions that result from specific genetic or infectious causes are exemplars of disease; for instance, cystic fibrosis and pneumonia. However, many medical syndromes are defined, not by their etiology, but by failures of functional systems that may have diverse etiologies. For instance, expressive aphasia results from damage to Broca's area that may have many possible causes. The search for similar specific genetic, neurophysiologic or anatomic abnormalities to explain bipolar disorder, major depression and schizophrenia has been disappointing, at best. It must proceed; specific causes will be found for some disorders. However, other possibilities have been neglected.

Some mental disorders may, like congestive heart failure (CHF), arise from failures of functional systems at higher levels of organization, failures that can have many different causes. Nosological concerns for CHF are minimal because heart failure can be measured objectively, and the physiology is well understood. The causes of a mental disorder may not only be multiple, they may arise from interactions among brain circuits and psychological mechanisms at several levels.

It is easy to see how blood sugar is stabilized by insulin secretion in response to high glucose levels. Understanding the functions of behavioral systems is harder. The mechanisms that regulate self-esteem, mood and anxiety are not susceptible to exactly parallel analyses at the cellular level because they are distributed among brain circuits and psychological mechanisms at several levels. For instance, managing social status requires processing innumerable cues in light of recalled prior information. A demeaning comment that begins to arouse resentment may be followed by a wink that changes the meaning into a shared joke--unless the individual's brain/mind is prone to paranoia.

Psychiatric nosology is constrained by the lack a functional understanding of normal behavior akin to what physiology provides for bodily functions. Behavioral ecology provides the closest comparable framework. It explains behavior in terms of its functional significance and effects on reproductive success, explanations essential in addition to those based on mechanisms [55]. For instance, it explains foraging behavior in terms of the costs and benefits of alternative strategies. It explains attachment in terms of its effects on fitness of infant and mother. This allows analysis of variations in attachment patterns--ambivalent, avoidant, and secure--as alternative strategies with costs and benefits in different situations [56]. The field is often called "evolutionary behavioral ecology" because such explanations are based on how selection shapes brain and psychological mechanisms that regulate behavior in ways that maximize Darwinian fitness [57].

Early applications of behavioral ecological are proving useful. Eating disorders may arise from dysregulation of systems that regulate food intake [58,59]. Syndromes arising from failures of attachment have been studied in detail [56]. Low self-esteem and narcissism may arise from dysregulation of status competition behaviors [60,61]. Mood disorders can be understood as disruptions in the system that adapts individuals to situations that vary in propitiousness [49]. Jealousy and diverse related symptoms may arise from the mechanisms that regulate mate competition and relationship maintenance [62,63]. Understanding dysregulation in these systems in behavioral ecological terms is not a substitute for understanding its causes in an individual, but it does offer an approach to the understanding of normal functioning somewhat parallel to what physiology offers to the rest of medicine [64,65].

The findings in some mental syndromes cohere, not because they come from a common etiology, but because they arise from failure or dysregulation of a functional system, or because they are responses often associated with a common situation, such as being in an abusive marriage. This suggests that some complaints about the comorbidity and heterogeneity of DSM diagnoses may arise from unrealistic expectations. There is no reason to expect that syndromes arising from dysregulated systems will have specific causes or sharp boundaries, and no reason to expect that a brain-based diagnostic system will ever be able to categorize them adequately. The comorbidity, heterogeneity and blurry boundaries of many DSM categories may accurately reflect clinical reality.

### Disorders from control system failures

Disorders are called "functional," if they arise from abnormal function of a system despite the lack of identifiable tissue abnormalities. Some, such as essential tremor, have observable clinical signs. Others, such as tinnitus, dizziness, fatigue, headaches and chronic pain, may have only subjective manifestations. Instead of specific cellular pathology, such disorders may be caused by feedback dysregulation at high levels of organization.

Vicious circles resulting from positive feedback at macro levels are responsible for many disorders. For instance, appendicitis is initiated by inflammation that compromises circulation at the neck of the appendix. This decreases ability to control infection, resulting in more infection, causing more inflammation and further compromise of circulation, in a cycle that escalates until the appendix bursts. On a slower time scale, osteoporosis may cause pain that limits exercise and results in additional bone loss.

Panic disorder may also result from positive feedback [66,67]. In patients concerned about their health, slight changes in heart rate and breathing cause fear, which causes additional physiological arousal, which further increases fear, in a spiral that escalates into a panic attack. A full explanation requires understanding individual differences in brain and cognition that make some people vulnerable to current situations arousing anxiety and the positive feedback cycle at the levels of cognition and emotion [68].

Cybernetic explanations may also help to explain other mental disorders [69]. Does dieting cause binging, which arouses greater fear of obesity and more intense dieting? Does depressive withdrawal from social life cause increased depression and further withdrawal? Does suspicion cause odd behavior, which results in whispered gossip, causing escalating suspicion, and increasingly odd behavior, and further whispering that arouses more suspicion? Disorders arising from positive feedback spirals are not likely to have disorder-specific neurophysiological changes. Their typical characteristics may be associated, not because they have a common cause or because they arise from a consistent brain abnormality, but because they are interacting aspects of a feedback cycle.

Do some mental problems arise at the level of information processing? Software problems can crash a computer even if the hardware is normal. If a program goes into an infinite loop or reaches a dead-end, the system will fail, even if every chip and connection is intact. If some mental disorders arise from analogous failures, we need to look for biomarkers in information systems. The analogy of minds with computers is far from perfect. Software is designed by engineers who create modules with specific functions. The programs they write have limited redundancy, so failure at any line of code may crash the program. Brains/minds are different; they are best understood using an entirely different metaphor (such as "wetware"). Because they were shaped by natural selection among miniscule variations over eons of time, their modules are less discrete, and they have myriad redundant interconnections and remarkable robustness. That a child can grow up to function almost normally after early removal of an entire brain hemisphere illustrates just how different brains are from computers. Nonetheless, it is worth considering the possibility that mental pathology can arise at the level of information processing.

## Conclusions

The search for satisfactory categories for mental disorders has been frustrating, at best. Hopes that DSM-III and IV diagnoses would map well to clinical and neuroscience realities have been dashed by studies that reveal a landscape charitably described as untidy. The disappointment is like that of immigrants who expect to find a city with streets of gold but who instead discover a chaotic jumble of muddy ruts.

The disappointment was magnified because the discrete categories of the DSM-III combined with wishes to emulate the rest of medicine to encourage a tacit narrow medical model that assumes disorders can be crisply defined by their causes, and that each disorder will have corresponding specific biomarkers. The actual model used in the rest of medicine is broader. Diagnostic categories are based on etiology when possible, but many are based on a physiological understanding of the normal functions of bodily systems. This broader medical model encourages separating symptoms that are protective responses from problems that arouse them. It also allows recognizing syndromes that reflect failures of functional systems that can have many causes, and functional syndromes that arise from dysregulation of otherwise intact systems. In short, psychiatry has been hoping to find disorders more discrete than many in the rest of medicine.

The difficulties are further magnified because psychiatry lacks the framework for understanding normally evolved functions that physiology provides for the rest of medicine. This makes it difficult to recognize the utility of protective responses, and to recognize syndromes that arise from failures of adaptive systems. Without the evolutionary/functional perspective the rest of medicine relies on to recognize syndromes, such as CHF, research in psychiatry has tended to look for causes at the levels of cells and molecules.

Unfortunately, describing functional systems that regulate behavior is not only in the early stages, it may also be intrinsically difficult. Evolutionary behavioral ecology and evolutionary approaches to psychology offer starting points, but behavior regulation systems do not just maintain homeostasis, they process thousands of bits of internal and external information in the light of prior experience, and current goals and strategies, to give rise to emotions and behaviors that tended to maximize reproductive success in ancestral environments. While it is now clear that these systems are nothing like a *tabula rasa*, it is increasingly obvious that they are also nothing like the components of a machine. They are not even as distinct as the components of other functional biological systems. The functions and localization of the loop of Henle, the mitral valve and glucose regulation are far more specific than those for motivation, memory or theory of mind. Despite these difficulties, opportunities abound. Mental disorders will be fully understood only when we can, as in the rest of medicine, understand pathology in terms of normal functions as well as normal mechanisms.

In the meanwhile, dissatisfaction with DSM categories may be tempered by encouraging more realistic expectations. Instead of specific diseases with specific causes, many mental problems are somewhat heterogeneous overlapping syndromes that can have multiple causes. Most are not distinct species like birds or flowers. They are more like different plant communities, each with a typical collection of species. Distinguishing tundra from alpine meadow, arboreal forest and Sonoran desert is useful, even though the categories are not entirely homogenous and distinct. Many mental disorders are similarly useful constructs, even if they frustrate the craving for reified categories with sharp boundaries defined by necessary and sufficient conditions.

## Abbreviations

CHF: congestive heart failure; DSM: Diagnostic and Statistical Manual of Mental Disorders; RDoC: Research Domain Criteria.

## Competing interests

The authors declare that they have no competing interests.

## Authors' contributions

Both authors contributed equally to all aspects of preparing this manuscript. They both read and approved the final manuscript.

## Pre-publication history

The pre-publication history for this paper can be accessed here:

http://www.biomedcentral.com/1741-7015/10/5/prepub
